# Prioritizing Indecent Image Offenders: A Systematic Review and Economic Approach to Understand the Benefits of Evidence-Based Policing Strategies

**DOI:** 10.3389/fpsyg.2021.606731

**Published:** 2021-02-09

**Authors:** Susan Giles, Laurence Alison

**Affiliations:** Department of Psychology, Institute of Population Health, University of Liverpool, Liverpool, United Kingdom

**Keywords:** indecent images of children, child sexual abuse, child sexual abuse material, risk assessment, arrest prioritization, police decision making

## Abstract

In 2013, there were an estimated 50,000 individuals involved in downloading and sharing indecent images of children (IIOC) in the United Kingdom (UK). This poses challenges for limited police resources. We argue that police officers can make most effective use of limited resources by prioritizing those offenders who pose the greatest risk of contact offending, by nature of demonstrable pedophilia, hebephilia or dual offending status and thus, those at highest risk must be dealt with first. What is currently lacking is a clear idea of the potential scale of the problem in socio-economic terms and why, therefore, it is so important that evidence-based approaches to offender detection and investigation continue to be a top priority for funders and policy makers. A systematic literature review was undertaken to address two related questions. First, what is the scale of the problem in the UK, in terms of the number of pedophilic and hebephilic individuals who pose a risk of contact offending against a child? Second, what is the potential socio-economic burden generated by the national IIOC suspect pool if left unattended to by targeted police action? Applying population estimates of pedophilia and hebephilia to the male population (16–89 years), we estimate there are between 2,365–5,991 males with paedophila and 12,218–30,952 males with hebephilia who are likely contact offenders. Applying average prevalence and incidence based costing methods to a conservative estimate of one victim per offender, the combined socio-economic burden from these persons could amount to £236-£597 million (incident costs) increasing to £2.9-£7.3 billion (lifetime costs; £3.3-£8.3 billion including QALY measures). Applying the same costs to CEOP (2013) estimate of 50,000 IIOC offenders we estimate that between 6,000 and 27,500 dual offenders could have already committed past contact offenses, contributing an economic burden of between £97–£445 million (incident costs) increasing to £1.2–£5.4 billion (lifetime costs; £1.4–£6.2 billion including QALY measures). Future contact offenses could contribute a further burden of £16–£18.6 million (incident costs) increasing to £198–£227 million (lifetime costs; £226–£260 million including QALY measures). Drawing upon these findings, we argue for the benefits of a research-informed prioritization approach to target IIOC offenders.

## Introduction

The number of indecent image of children (IIOC) offenses, in recent years, has created a growing workload for the police officers investigating them. Convictions for IIOC offenses are increasing (Sentencing Council, 2012) and police forces are struggling to manage the demand and risks posed by this offending group. In the United States of America (USA), for example, Waters testified to United States Congress that despite 500 000 national offenders being identified, only 2% of cases were being investigated due to a lack of police resources (Committee on the Judiciary, 2008). He described the situation as “catastrophic” as the magnitude of the problem had already overwhelmed USA law enforcement's forensic and investigative infrastructure. In the United Kingdom (UK), the Child Exploitation and Online Protection Center (CEOP, 2013) received 8,000 reports in 2012 that contained a total of 70,000 still and moving IIOC. This represents a two-fold increase from the previous year. They estimated that in 2012, there were around 50,000 individuals involved in downloading and sharing IIOC in the UK (CEOP, 2013). More recently, 39 police forces recorded 17,521 obscene publication offenses against children in the year ending March 2019 and identified 552 unique victims within IIOC (Elkin, 2020). These figures help to provide some indication of the size of the task now faced by British and international investigating authorities.

Safeguarding children is at the forefront of the policing agenda; with the aim of protecting children from abuse in the first place (where possible) and reducing the likelihood of reoffending and repeat victimization. CEOP (2013) claim that 20% of the images they received in 2012 had been assessed as being self-generated by offenders; raising concerns about contact offending and the need to identify and safeguard these children. Set against this, meta-analysis by Seto et al. (2011) confirms that there is a considerable subgroup of offenders who offend online only (by downloading and sharing images) and who are unlikely to present a risk of contact offending. This article argues that evidence based policing approaches can help prioritize those offenders at highest risk of committing contact offenses and in so doing, demonstrate the most efficient use of limited public sector resources toward safeguarding goals.

Seto et al. (2011) found approximately one in six internet offenders are expected to have a history of contact sexual offenses. Criticality is important here; researchers report substantial variation in reported prevalence rates dependent upon what method is used. Prevalence rates reduce to one in eight when using official data and increases to one in two when using self-reports. As Seto et al. (2011) meta-analysis is primarily based on studies involving official data it is likely that a prevalence rate of one in six is also an underestimate. These figures do not take account of undetected offenders. An analysis of undetected offenders by Neutze et al. (2012) suggests a higher rate of undetected dual (offline and online) offending than would be suggested by Seto et al. (2011) meta-analysis. Research examining specialization and “cross over” (Howard et al., 2014) suggest that IIOC offending does not tend to lead to contact sexual offenses but that the converse is not true; specialization is most evident for IIOC offenders but contact sexual offenders against a child will “cross over” to IIOC offending. Further, frequency of pornography use (including offline IIOC) has been shown to contribute to the prediction of crime and violent (including sexual) recidivism for child contact sexual offenders (Kingston et al., 2008). This suggests a general paraphilic lifestyle for some high risk dual offenders and that police resources should be focused on dual offenders. Set against this, there is still a recognized risk that those with IIOC offenses may cross over to contact offending. In their meta-analysis, Babchishin et al. (2014) propose that the psychological factors that differentiate those who do and do not act on their interests should be related to propensities for rule violation. Those most at risk for cross over offenses would be expected to have high levels of pedophilia, high levels of anti-sociality, have access to children, and have few psychological barriers to acting on their deviant impulses. Those least at risk would experience the converse. These findings provide useful insights into potential decision criteria, although a limitation of Babchishin et al. (2014) meta-analysis is that up to half of IIOC offenders could be expected to have undetected offline offenses (Seto et al., 2011). As such, the profile of IIOC offenders is recognized as diverse and the management of offenders with IIOC offenses should carefully consider the existence of concomitant contact sexual offenses.

The scale of the IIOC sex offending problem, both now and in the long term, has prompted multidisciplinary research in the area, with collaborations between academics and police forces to help improve detection and direct the allocation of policing resources to the most concerning cases. Researchers such as Steel (2009), Hughes et al. (2006) Brennan, Hammond and colleagues (Hammond et al., 2009; Brennan and Hammond, 2011, 2017) are beginning to explore the investigative and inferential value of examining pedophilic-related search terms and other online behavior amongst IIOC offenders. Hammond et al. (2009) and Brennan and Hammond (2017) for example, suggest that there is evidence of quite discrete paraphilic sexual interests to be found in offenders' online behavior. Their analysis of 119,869 search terms and 3,000,000 records (representing one week of activity on a P2P site) revealed seven class taxonomy of paraphilic use; indicating the presence of distinct paraphilic sub-communities in the P2P network space (Hammond et al., 2009). This included pedophilic, hebephilic, gerontophilic, bestiality, sadistic, rape and incest sub-communities. The concern here is that whilst there is a wide range of offenses, pedophilic individuals are at increased risk of contact offenses against children. Research has demonstrated the serial nature of pedophilia, the large number of children abused by each pedophilic individual and the under reporting of assaults (e.g., Abel and Harlow, 2001; Hall and Hall, 2009). Hammond et al. (2009) suggest that effective targeting strategies, such as those that draw upon the action of distinct online sub-communities, may be a more effective strategy for targeting offenders than a random or “scatter gun” (pg. 7) approach.

An alternative approach espoused by the Fighting International Internet Pedophilia (FIIP) project, a collaboration between Kent Police and academics at the University of Liverpool, is to prioritize the investigation of known IIOC offenders who share features with dual offenders (those who have committed IIOC and contact offenses). This approach has led to the development of the Kent Internet Risk Assessment Tool (KIRAT: Long et al., 2016). KIRAT does not purport to predict future risk or re-offending. It is a four-tiered decision tool used nationally by UK Police to prioritize IIOC offenders, based on nine features most often associated with dual offending, with upwards of 95% accuracy for higher risk offenders and <20% false positive rates for lower risk offenders. Recent cross-cultural validations have successfully demonstrated the relative homogeneity of KIRAT risk factors for contact offending across 24 countries. A further recidivism tool (CPORT) with investigative value has also been developed by Michael Seto and colleagues (Seto and Eke, 2015; Eke et al., 2019) and has recently been applied in a Spanish context (Soldino et al., 2019).

The approaches outlined above demonstrate that it important to have a conceptually driven and empirically supported approach about the links between online behavior and risk, which guides practical action. Whilst lower risk cases still need to be processed through the legal system (Long et al., 2016) police officers can facilitate optimal attainment of harm reduction goals by prioritizing those offenders who pose the greatest risk of contact offending, by nature of demonstrable pedophilia or dual offending status and thus, those at highest risk must be dealt with first. A random approach would arguably be less effective beyond the general deterrence that it may provide.

What is currently lacking is a clear idea of what the potential scale of the problem is in socio-economic terms. Child sexual abuse victimization impacts society (in terms of costs of supporting victims) along with the long term economic development of victims (in terms of mental well-being, education and employment). Economic metrics that seek to establish the disease burden associated with child sexual abuse encapsulate this interdependence by establishing costs to society along with the less tangible emotional costs to victims. As such, we prefer the term socio-economic to describe our approach. By establishing the scale of the problem in socio-economic terms we aim to demonstrate the importance of evidence-based approaches to offender detection and investigation and that they should continue to be a top priority for funders and policy makers. The aim of the current article is to expand and develop upon this debate by drawing on published prevalence studies and econometrics to present some of the economic burden that could be contributed by IIOC offenders. It draws on a systematic review and economic approach to examine two research questions. First, what is the potential scale of the problem in the UK, in terms of the number of and socio-economic burden of males with pedophilia and hebephilia who pose a risk of contact offending against a child? Second, what is the socio-economic burden potentially generated by the national suspect pool of 50,000 IIOC offenders?

## Materials and Methods

The broad objective of the work is to estimate the potential scale of the problem in the UK by establishing offending and economic figures that could form the basis of sensible cost estimates. As the body of literature has not been comprehensively reviewed for these purposes, a scoping review was preferred. This would allow a preliminary assessment of the size and scope of the existing literature and the extent to which it could be used to answer the two research questions. Where gaps in knowledge are identified, we can draw on the available evidence to make informed decisions about derived estimates. As such, neither narrative nor quantitative synthesis were the primary aims of this paper. Quality assessment and data extraction that you might anticipate from systematic review or rapid evidence assessment was therefore, limited. Rather, we follow in the economics tradition; producing a systematic literature review to help provide an overview of the available evidence from which we make critical decisions about which figures to use in our cost estimates. The estimates that we provide should be seen as a “starting point” of a much wider discussion about the benefits of police action in this area and how these benefits may be measured. Toward this end, the first author undertook a systematic literature review in August 2020, to identify relevant academic and gray literature that would inform our research questions.

### Search Strategy

The search strategy included setting objectives and specifying inclusion and exclusion criteria for reviews, designation of search terms and databases to be searched. The following databases were examined for published material 2000–2020; Cochrane library, Web of Science, Scopus, PubMed, APA PsychINFO and National Criminal Justice Reference System (NCJRS). There followed conduct of searches, de-duplication, application of screening procedures and compilation of target lists for further review. Material would be excluded if full text was unavailable in English, or unavailable through the University of Liverpool online library services. Where possible, the search results were exported into reference management software, *Endnote Online*. Search teams were applied to online searches (Google/Google Scholar) to identify soft literature available in the public domain. Decisions were made by the research team to select economic and prevalence figures that would best provide a sensible basis for cost estimates. In the discussion that follows we provide an account of our search strategy, the relevant material that was found along with a critical discussion of that material that led to our decision about which material (prevalence, contact offending, economic figures) to include in our cost estimates.

### Estimating Victim Costs

A key objective of the review was to estimate the average victim cost for child sexual assault including children who are sexually assaulted during the commission of a first generation IIOC offense (the offender records the abuse). If arrest prioritization tools are effective by picking out contact offenders then these costs to the victim and the National Exchequer can be averted; constituting costs saved for future contact victims, Historical victims can also be safeguarded from repeat victimization.

Material would be included if it provided an economic analysis of the impacts of sexual harm that could be extracted directly from the published account. Econometrics emerging from sexual assaults against children and adults were both considered on the basis that the latter is a more established field of study and could shed light on costs experienced by child victims. Material would be excluded if it did not provide unit level costs (i.e., costs per victim). The first author applied Boolean operators to search terms to generate search phrases (cost AND victim AND sex*crime OR rape OR child sex*abuse OR indecent image* OR internet sex* offend*OR online sex*offend OR contact sex often OR groom* OR chat room off* or solicitation off* OR molest* OR pedo* OR paedo*), resulting in 523 items. These items were then screened for filtering, such that items were excluded based on duplication, or on the basis that the full text did not inform the research question. Subsequent screening of 50 items was undertaken by the first author. A great wealth of material was available around the impact of sexual harm on victims, the relative costs of reporting and non-reporting of sexual abuse, and factors affecting disclosure of abuse. Promising work is beginning to emerge around the vicarious costs associated with being a victim of an IIOC offense. However, this work is arguably too underdeveloped for the purposes of the present paper as it relies on US legal case studies or potential restitution frameworks (Jacques, 2011; Cassell et al., 2013). As such, these references were excluded. Twenty three references provided unit costs for victims of sexual assault, including 8 that provided a focus on child abuse or child sexual abuse. Of the remaining 23 references, 3 were discarded on the basis that more recent updates were available [i.e., Heeks et al. (2018) was retained whilst Brand and Price (2005), Dubourg et al. (2005) and Home Office (2011) were discarded]. Table 1 provides a summary of the remaining 20 references. These references draw upon a range of economic and survey methods to establish sensible (and often conservative) cost metrics. Three broad costing methods are employed. Incident methods attempt to identify the cost per incident and can range in scope from single costs (e.g., cost of medical or mental health treatment) to total costs incurred. Prevalence methods draw out incident costs but examine the number of cases reported within a year period to establish the total cost of disease incurred that year. Incidence methods calculate the lifetime costs of cases first diagnosed in a particular year. The references in Table 1 vary in scope, but provide rich information on the range of tangible and intangible costs attributable to sexual harm. Tangible costs are often more readily quantifiable; they include those costs that arise immediately or proximally to the offense, such as medical care, mental health services, criminal justice costs, victim/children's services and loss of economic productivity. Intangible costs are the hardest to quantify; constituting the human and emotional cost to victims. A range of measures are employed (quality of life years adjusted, jury compensation awards and willingness to pay) to try capture meaningful metrics on the reduced quality of life resulting from victimization. In Table 1 half of the references are solely focused on quantifying tangible costs, with seven incorporating intangible costs and three focused specifically on the issue of intangible costs and how they can be best be measured. For this research it was deemed important to select a study that incorporated both tangible and intangible costs. From these, Saied-Tessier (2014), Letourneau et al. (2018) and Heeks et al. (2018) were considered for inclusion in further economic analysis given their focus on either child sexual abuse, the full range of costs included and/or focus on the United Kingdom (UK).

**Table 1:** Unit costs of sexual abuse and child sexual abuse (published 2000–2020).

**References**	**Country**	**Offense type^a^**	**Costing method^b^**	**Security**	**Health**	**Criminal justice**	**Victim services**	**Labor**	**Intangible**	**Unit cost**
Cohen et al. (2004)	USA	SV	WTP						✓	US resident willing to pay $126 per year for 10% reduction in rape and sexual assault
Dolan et al. (2005)	UK	SV	P		✓				✓	Monetary value of discounted QALY losses £16,480 rape and £4,790 sexual assault. Intangible victim costs £1,027 rape and £341 sexual assault
Fang et al. (2012)	USA	CA	I		✓	✓	✓	✓		Lifetime cost for non-fatal child maltreatment $210,012
Gelles and Perlman (2012)	USA	CA	P		✓	✓	✓	✓		National prevalence provided
Heeks et al. (2018)	UK	SV	P	✓	✓	✓	✓	✓	✓	Rape £39,360; and other sexual offenses £6,520
Hunt et al. (2017)	USA	SV	In			✓				Average cost to taxpayers for legal services per rape/sexual assault is $2000-$5000
Letourneau et al. (2018)	USA	CSA	I	✓	✓	✓	✓	✓	✓	female victim of non-fatal CSA $282,734; male victim of non-fatal CSA
McCollister et al. (2010)	USA	SV	In		✓	✓		✓	✓	Combined tangible and intangible cost per rape/sexual assault $240,776
New and Berliner (2000)	USA	CSA	In		✓					Average amount billed for mental health treatment was $2634.09
Olavarria-Gambi (2007)	Chile	SV	P		✓					$1,368.8 medical assistance to victims of rape and sexual assault
Peterson et al. (2017)	USA	SV	I		✓	✓		✓		Lifetime economic burden of rape per adult female $122,461
Peterson et al. (2018)	USA	SV	I					✓		$730 average cost of lost productivity across victims of intimate partner violence, sexual violence and stalking
Post et al. (2002)	USA	SV	P		✓	✓	✓	✓	✓	Cost of rape and sexual assault $94,466 1996 USD
Saied-Tessier (2014)	UK	CSA	P		✓	✓	✓	✓	✓	National prevalence provided
Shanahan and Donato (2001)	Australia	CSA	In		✓	✓	✓		✓	Tangible and intangible victim costs between $119,340-$218,790
Smith et al. (2014)	Australia	SV	P		✓			✓		Incident costs for sexual assault $3,912
Tennessee et al. (2017)	USA	SV	In		✓					$6,737 hospital billing for victims of sexual assault
Walby (2004)	UK	DV	P						✓	Rape and assault by penetration £104,300; and non-penetrative sexual assault £240
Wang and Holton (2007)	USA	CA	P		✓	✓	✓	✓		National prevalence provided
Yang et al. (2014)	USA	CSA	P		✓	✓	✓	✓	✓	Estimated costs averaged $159,610 *per sexual* violence incident for children, $110,937 rape of adult and $389 sexual assault of adult

a *CA, Child abuse; CSA, Child Sexual Abuse; DV, Domestic Violence; SV, Sexual Violence*.

b *I, Incidence; In, Incident; P, Prevalence; WTP, Willingness to Pay*.

Saied-Tessier (2014) provides a comprehensive review of psychological and physical health problems experienced by victims of child sexual abuse in her prevalence based assessment of child sexual abuse in 2014. She argues that costs per victim do not appear to exist for the UK and represents a much needed area of research. This NSPCC report focuses on annual costs and whilst unit costs are provided, where available, individual level data is arguably still too underdeveloped for the purposes of the present report. As such, Saied-Tessier (2014) was not utilized for further analysis. As such, a decision was made to calculate lower bound costs from the prevalence based (annual) costs provided by Heeks et al. (2018); (UK) and upper bound costs from the incidence based (lifetime) cost estimates provided by Letourneau et al. (2018) (United States).

Heeks et al. (2018) provide sexual offense unit costs of victims in the UK. The Home Office focus takes account of a wide range of child sexual offenses (such as intercourse with a female under 13 years/16 years, incest and indecent assault against a minor) in its definition of sexual offenses and the figures below can be applied to cases of child sexual abuse. However, the total burden costs are based on responses from adults (16–59 years old) to the Crime Survey for England and Wales. Adults complete a Self-Completion Module that asks questions about sexual abuse experienced during childhood. The Self Completion Module is not used in the 10–15 year old survey as it is too sensitive. As such, prevalence and economic figures do not specifically take into account offenses against children that have taken place 2015/16. There are limitations in applying adults cost to children though, arguably, it is a conservative strategy because children are likely to experience negative effects of child sexual abuse throughout their lifetime.

To provide a cost per incident, the figures from Heeks et al. (2018) were extracted and retained for subsequent cost estimates. Heeks et al. (2018) calculate the unit cost of rape as £39,360 and other sexual offenses as £6,520 in 2015/16. These figures increase to £43,214 and £7,158 in 2019 using Bank of England Inflation Calculator (Bank of England Inflation Calculator) online “inflation” calculator with inflation averaged at 3.2% a year. We do not know the exact extent to which child sexual abuse involves acts of penetration but we can estimate this from the most recent figures provided by the Office of National Statistics (2020)^1^. Around one quarter of child sexual offenses recorded by the police March 2018–2019 were rape offenses (ONS, 2020)^1^. Whilst one might argue this risks conflating figures as rapes might be more likely reported to the police than sexual assaults, responses to the anonymous CSEW Self Completion Module reported by ONS (2020)^1^ found that roughly one third of those reporting any contact sexual offense before the age of 16 had experienced rape and assault by penetration (including attempts). As such, in calculating incident cost estimates we conservatively estimate 25% offenses at £43,214 and 75% at £7,158.

Letourneau et al. (2018) provide average lifetime costs for victims of non-fatal child sexual abuse in the USA in 2015. Drawing upon a wider range of measures than Heeks et al. (2018) such as education costs, they also draw on the available child sexual abuse literature to establish life time effects on health, criminal offending, suicide death and QALY losses. They estimate that the lifetime cost for female victims of non-fatal child sexual abuse is $282,734. Estimates for male victims are “artificially low” (pg. 419) at $74,691 due to a lack of reliable data pertaining to losses in male victim work productivity. Similar to Saied-Tessier (2014), they calculate loss to quality of life separately from estimated total burden because of mixed opinion about the validity of converting QALY to a monetary amount (e.g., see Dolan et al., 2005). Separately, they estimate quality of life losses as $41,001 for female victims and $38,904 for male victims.

Converting these figures to the UK context required a number of decisions to be made. First, Letourneau et al. (2018) do not differentiate different forms of child sexual abuse and so it is not possible to estimate costs based on rape or sexual assault (as in Heeks et al., 2018). Set against this, Letourneau et al. (2018) draw on an established literature that examines the impact of child sexual abuse in general. As such, we were confident that their robust and replicable incidence based methods would not lead to inflated life time estimates. The literature on harms emerging from different forms of abuse is arguably too under developed to meaningfully change their estimates. Second, Letourneau et al. (2018) establish total burden based on 75% of victims being female. This is in spite of absent male work productivity data significantly impairing male estimates (apart from a small difference in suicide death costs all other costs are equal). In this paper, we decided that differences between males and females in Letourneau were methodological artifacts and so would not be applied. As such, we would apply the value provided for female victims. Third, their values are provided in 2015 USD. Examining exchange rates provided by HM Revenue Customs Exchange Rates (2011) the average value of $1 was £0.6255 (average for the year to 31st March, 2015). As such, $282,734 was calculated as £176,850 in 2015, this figure increases to £197,535 in 2019 using Bank of England online “inflation” calculator with inflation averaged at 2.8% a year. This would be used to estimate lower bound lifetime costs. Upper bound estimates would include quality of life losses adjusted to $40,477 to account for 75% of victims being female (as these figures were not affected by paucity of male productivity data). This was calculated as £25,318 in 2015 and increased to £28,279 in 2019 using Bank of England online “inflation” calculator with inflation averaged at 2.8% a year. As such, an upper bound estimate of lifetime cost would be £225,814.

### Estimating Prevalence of Pedophilia and Contact Offending

To support review question 1, a systematic search of the literature was undertaken to identify a) prevalence of pedophilia (interest in pre-pubescent children, generally aged 13 years and younger) and hebephilia (interest in pubescent children) in the general population, and b) rates of contact offending amongst pedophilic and hebephilic individuals.

In terms of prevalence, material would be included if it provided population-based prevalence rates that could be extracted from published accounts. Clinical and forensic samples were not retained. Community and convenience based samples were retained for further inspection. Since Seto (2008) provides an outline of prevalence studies published 2000–2013 the first author made use of these resources (of which five studies potentially met the study criteria) and searched for journal articles published post 2013. She applied Boolean operators to search terms to generate search phrases (“prevalence” AND “paedophil*” OR “pedophil*” OR “hebephil”), resulting in an additional 219 items. These items were then screened for filtering as described above. Seventeen items were downloaded for further consideration. Only 8 of these items provided a population based estimate of pedophilia or hebephilia with Santtila et al. (2010, 2015) drawing upon the same data set. Three additional post 2,000 references were identified from Seto (2018). Table 2 provides a summary of 11 references that met the inclusion criteria.

**Table 2:** Outline of prevalence studies (published 2000–2020).

**References**	**Country**	**Method**	**Sample**	**Prevalence**
Abdullahi et al. (2015)	Nigeria	Survey	Student: 871 randomly selected sample from one university (*n* = 447 males, mainly 18–25 years)	0.2% males reported pedophilia (symptoms lasted up to 6 months/causes distress)
Ahlers et al. (2011)	Germany	Survey	Community: 367 males in a metropolitan city (from a representative sample of 6,000 men aged 40–79 years)	9.5% had sexual fantasies about pre-pubescent child; 6.0% had masturbation fantasies; **3.8% had experienced sexual contact with a pre-pubescent child**
Bartova et al. (2020)	Czech Republic	Survey	Community: 10,044 stratified random online sample (*n* = 5,023 males, aged 18–88 years)	0.6% disclosed a sexual preference in pre-pubescent children (causing 3.3% to confide in a health care professional; 3.1% disclosed a sexual preference in pubescent children (causing 11.5% to confide in a health care professional; 0.9% sexually aroused by pre-pubescent children (4.4% pubescent); 0.6% disclosed “porn use” with respect to pre-pubescent children (1.7% pubescent); 0.4% experienced sexual fantasies about pre-pubescent children once a week/every day (1.4% pubescent); 1% would definitely have sex with a pre-pubescent child if it was legal (4.4% pubescent)
Dawson et al. (2014)	Canada	Survey	Student and online; 1,015 non-representative sample (*n* = 305 males, average age 23 years)	0.6% report sex with a pre-pubescent child as sexually arousing; 0.9% rated sex with a pubescent child as sexually arousing
Dombert et al. (2016)	Germany	Survey	Community; representative sample of 8718 males aged 18–89 years	Estimate upper limit of 5.4% non-exclusive paedophilic disorder, with 4.1% reporting sexual fantasies involving pre-pubescent children and 0.1% evidencing exclusive pedophilia; 3.2% indicated sexual behavior involving pre-pubescent children, with 1.7% indecent image offenders, 0.8% reporting sexual contact with children and 0.7% mixed offenders (**1.5% combined contact offending**); 0.4% had paid a child for sexual services, similarly 0.4% had intended child sex tourism
Joyal (2014)	Canada	Survey	Online; non-representative sample of 1,516 (*n* = 717 males)	1.8% ever fantasized about pre-pubescent children
Santtila et al. (2010, 2015)	Finland	Survey	Twin study: 1,310 males aged 33–43 years	0.2% reported sexual interest in children aged 12 or younger; 3.1% for children aged 13-15 years; combined 1 year incidence estimated at 3.3%
Seto et al. (2015)	Sweden	Survey	Schools; representative sample of 1,978 males aged 17–20 years	4.2% reported use of indecent images of children, which was positive associated with self-reported coercive sexual behavior involving pre-pubescent children; **14% reported sexually coercive behavior against a pre-pubescent child**
Williams et al. (2009)	Canada	Survey	Student; non-representative sample of 191 males (average age 19.7 years)	12% admitted any sexual fantasy about children; **5% admitted any sexual behavior involving children**
Wurtele et al. (2014)	USA	Survey	Student and online; 435 non-representative sample (*n* = 173 males, aged 18 years and older)	Nearly 10% reported some likelihood of having sex with children or viewing child pornography if they would not be caught or punished; 4% reported some agreement with each of following items, fantasize about having sex with child, being sexually attracted to little children, masturbation fantasies about sex with children

*Bold values indicate prevalence of contact offending provided in reviewed papers*.

American Psychiatric Association (2013) estimate that the highest possible prevalence for pedophilic disorder in the male population is ~3–5%. Seto (2008) agrees these as upper limits and has suggested more conservative estimates in recent years (1–3%; see Seto, 2018). The epidemiological problem stems from the inability to fully explore the diagnosis of pedophilia or hebephilia using survey methods. Studies, such as those outlined in Table 2, include self-report measures of sexual fantasies, arousal, viewing child-adult sex and to a lesser extent behavior but they do not typically explore persistence, recurrence nor intensity of such thoughts, feelings and behaviors (Seto, 2018). As such, prevalence is likely to be overestimated. That said, one could also argue there are issues with self-disclosure and that these surveys identify only those individuals willing to admit paraphilic thoughts. An alternative view expressed in the literature is that surveys such as these might attract individuals who are more sexually curious, open-minded or deviant (Ahlers et al., 2011; Santtila et al., 2015). Whilst recognizing these limitations, the prevalence rates described in Table 2 were considered for inclusion in economic analysis.

Three studies were considered for inclusion based on (a) their focus on a wider range of ages, (b) large sample size and their attempts to identify a representative sample and/or (c) attempt to differentiate prevalence of pedophilia and hebephilia. In their stratified random online sample of the Czech population, Bartova et al. (2020) report a *current* sexual preference for pre-pubescent children amongst 0.6% of males (aged 18–88 years) and for pubescent children amongst 3.1% of males. This is similar to Santtila et al. (2015) who in their attempt to identify a *1 year incidence* of pedophilia in their analysis of 1,310 Finnish twins report a prevalence of 0.2% for pedophilia (children aged under 12 years) and 3.1% for hebephilia (13–15 years). The age range of their study is more limited, focused on males aged 33–43 years. In their online survey of 8,718 German men, Dombert et al. (2016) explore *lifetime* sexual interest in pre-pubescent (children aged under 12 years) only, reporting 0.1% exclusive pedophilic sexual preference and an upper estimate of 5.4% non-exclusive pedophilic sexual interest. Though the findings from these three studies are broadly similar, we decided to opt for Bartova et al. (2020) for a number of reasons. First, both Santtila et al. (2015) and Dombert et al. (2016) define paedophila as a sexual interest in children under 12 years. This makes sense given many children biologically mature earlier than 13 years, however, this does not match the cut off criterion for prepubescence outlined in DSM V nor does it align with UK legislation (the Sexual Offenses Act 2003 uses distinct differences between sexual assaults on children, section 5–8 specifically distinguishes “rape and other offenses against children under 13 years”). Like Dombert et al. (2016) and Bartova et al. (2020) explore a wider range of ages than Santtila et al. (2015) but compared to the former Bartova et al. (2020) provide separate estimates for pedophilia and hebephilia. As such, we proceed with the estimate that 0.6% of the UK male population exhibit pedophilic sexual preferences and 3.1% exhibit a hebephilic sexual preference.

It is difficult to estimate the number of pedophilic and hebephilic individuals that pose a risk of contact offending as this is not always explicitly considered in research. Bartova et al. (2020), for example, omitted to ask about contact offending because of its criminal implications, preferring to ask whether respondents would engage in such behavior if it were legal. Four studies in Table 2 had attempted to address the issue of contact offending in their prevalence studies. Sexual contacts with children range from 1.5 to 14% with the larger prevalence figure exploring a broader range of sexually coercive behaviors amongst young adults (Seto et al., 2015). Ahlers et al. (2011) and Dombert et al. (2016) provide representative samples from a wider range of ages than Seto et al. (2015) and Williams et al. (2009) and so were considered for inclusion. In line with the purposes of the current paper, Ahlers et al. (2011) attempt to extrapolate self-reported rates of contact offending to the general population. They apply observed prevalence (14/376) but use the number of individuals invited to participate in the research as the denominator (14/6,000) resulting in a much more conservative contact offending prevalence rate of 0.23% (rather than 3.8% of the sample). This translates to 1,800 and 69,000 males (aged 40–79 years) who have acted out their sexual interest on pre-pubescent children in the City of Berlin and Germany, respectively. They do acknowledge that the absolute number is likely to be considerably higher. Their figures are based on self-disclosure amongst men aged 40–79 years, whereas they acknowledge that 20-40 year olds are both sexually and criminally more active. Further, only non-exclusive heterosexual pedophilic types appeared to participate in Ahlers et al. (2011) and so it is unclear how well this prevalence would translate to exclusive nor homosexual pedophilic types. Dombert et al.'s (2016) sample includes those with an interest in males and females, they have also used a particularly in-depth methodology more closely aligned with DSM V. As such, we decided to use Ahlers et al.'s (2011) 3.8% as an upper bound estimate and Dombert et al.'s (2016) 1.5% prevalence as a lower bound estimate of contact offending. Note, neither of these references provide estimates of contact offending for hebephilic individuals. Further, we elected to use self-reported prevalence from the sample rather than the population-based sample approach taken by Ahlers et al. (2011). There are many reasons why the remaining 5,624 individuals did not participate in their study; no doubt ranging from repulsion to undisclosed contact offenses.

### Past and Future Offending of IIOC Offenders

Since Seto and colleagues have already conducted a detailed meta-analysis on past and future offending of IIOC offenders (Seto et al., 2011) we did not conduct further systematic search of the literature. In terms of past offending, Seto et al. (2011) found approximately one in six internet sex offenders have a history of contact sexual offenses. Prevalence rates reduce to one in eight (12%) when using official data and increase to one in two when using self-reports (55%). In line with Seto et al. (2011) findings we adopt 12 and 55% as lower and upper bound risk estimates for past contact sexual offending. In terms of reoffending, recidivism studies (Seto et al., 2011; 1.5–6 year follow up) indicate that internet sexual offenders have a lower rate of sexual recidivism (4.6%) than contact sexual offenders (e.g., 13.7%). Where information is available on type of sexual recidivism in Seto et al. (2011) meta-analysis, 2% recidivate with a contact sexual offense and 3.4% recidivate with a further IIOC offense. These figures are likely to be underestimates as information is based on official criminal records in all samples.

On the balance of evidence, dual offenders demonstrably show slightly higher rates of both internet and contact sexual offenses than internet sexual offenders (Goller et al., 2010; Graf and Dittmann, 2011; Wakeling et al., 2011). As Wakeling et al. (2011) study provides an analysis of a large UK data set–a “routine” correctional sample (*N* = 1,344 UK IIOC offenders), we elected to use this study to help inform upper bound estimates for dual offenders. Wakeling et al. (2011) found that dual offenders had a two year proven sexual reoffending rate of 6.6% whilst internet specialists had a reoffending rate of 1.6%. In the economic analysis that follows we draw on both Seto et al. (2011) and Wakeling et al. (2011) to provide lower and upper bound reoffending estimates.

## Results

### Review Question 1: What Is the Potential Scale of the Problem in the UK, in Terms of the Number of and Socio-Economic Burden of Pedophilic and Hebephilic Individuals Who Pose a Risk of Contact Offending Against a Child?

Applying Bartova et al. (2020) prevalence rates of 0.6% for pedophilia and 3.1% for hebephilia to mid-year population estimates 2019 (*www.ons.gov.uk*) of adult males (16–89 years) in the UK (*N* = 26,275,165), there could be as many as 157,651 males with pedophilic sexual preferences and 814,530 males with hebephilic sexual preferences. To get a sense of those that pose a risk of contact offending we apply Ahlers et al.'s (2011) prevalence of 3.8% as an upper bound estimate and Dombert et al.'s (2016) prevalence of 1.5% as an lower bound estimate of contact offending. The estimated number of contact offenders multiplied by incident and lifetime victim costs are provided in Table 3.

**Table 3:** Estimated socio-economic burden attributable to paedophilic and hebephilic contact sexual offending (one victim per offender).

**Population**	**Contact offending prevalence**	**Contact offenders (*n*)**	**Incident costs^a^**	**Lifetime costs^b^**	**Lifetime costs (with QALY)^c^**
Males with pedophilia (*n* = 157,651)	Lower bound: 1.5% (Dombert et al., 2016)	2,365	£38,237,766	£467,170,275	£534,050,110
	Upper bound: 3.8% (Ahlers et al., 2011)	5,991	£96,895,466	£1,183,432,185	£1,352,851,674
Males with hebephilia (*n* = 814,530)	Lower bound: 1.5% (Dombert et al., 2016)	12,218	£197,589,496	£2,413,482,630	£2,758,995,452
	Upper bound: 3.8% (Ahlers et al., 2011)	30,952	£500,555,744	£6,114,103,320	£6,989,394,928

a *Estimate 25% offenses at £43,214 (penetrative) and 75% at £7,158 (non-penetrative) in 2019 GBP*.

b *Estimated at £197,535 in 2019 GBP*.

c *Estimated at £225,814 in 2019 GBP*.

We estimate that there could be between 2,365 and 5,991 males with pedophilia who are likely contact offenders. Applying Heeks et al.'s (2018) costs of £43,214 (25%) and £7,158 (75%) this could mean between £38 million and £97 million socio-economic burden (based on a conservative estimate of one victim per offender). Applying life time costs this could increase to £467 million (£534 million, QALY) to £1.2 billion (£1.35 billion, QALY). Applying the same assumptions to population estimates of males with hebephilia, we estimate between 12,218 and 30,952 males are likely contact offenders, translating to between £198 million and £500 million incident costs and between £2.4 billion (£2.8 billion, QALY) and £6.1 billion (£7 billion, QALY) life time costs.

### Review Question 2: What Is the Socio-Economic Burden Potentially Generated by the National Suspect Pool of 50,000 IIOC Offenders?

#### Historical Victims Are Potentially Identified and Safeguarded

In line with Seto et al. (2011) findings we adopt 12 and 55% as lower and upper bound risk estimates for past contact sexual offending. Economic estimates are outlined in Table 4.

**Table 4:** Estimated socio-economic burden attributable to historical contact offending of IIOC suspects (estimated at one victim per offender).

**IIOC suspects (*N* = 50,000)**	**Offending prevalence**	**Contact offenders (n)**	**Incident costs^a^**	**Lifetime costs^b^**	**Lifetime costs (with QALY)^c^**
Past contact offending	Lower bound: 12% (Seto et al., 2011)	6000	£97,032,000	£1,185,210,000	£1,354,884,000
	Upper bound: 55% (Seto et al., 2011)	27,500	£444,730,000	£5,432,212,500	£6,209,885,000

a *Estimate 25% offenses at £43,214 (penetrative) and 75% at £7,158 (non-penetrative) in 2019 GBP*.

b *Estimated at £197,535 in 2019 GBP*.

c *Estimated at £225,814 in 2019 GBP*.

##### Lower Bound Estimate Based on Official Records

It would be expected that at least 6,000 individuals in the UK who download and trade IIOC online will have an official history of contact sexual offending. Applying the average cost per victim (incident, lifetime and lifetime plus QALY) to a conservative estimate of one prior victim per offender, the potential UK suspect pool *could have already* contributed toward a socio-economic burden between £97 million (incidence costs) and £1.2 billion-£1.35 billion (QALY)(lifetime costs).

##### Upper Bound Estimate Based on Self-Reports

It might be expected that as many as 27,500 would disclose a history of contact sexual offending (including undetected cases). Again, applying the cost per victim of sexual assault to a conservative estimate of one prior victim per offender, the potential UK suspect pool *could have already* contributed an economic burden between £445 million (incident costs) and £5.4 billion–£6.2 billion (QALY) (lifetime costs).

#### Potential Future Victims May Be Safeguarded

Estimates associated with future offending are provided in Table 5.

**Table 5:** Estimated socio-economic burden attributable to future offending of IIOC suspects (estimated at one victim per offender).

**IIOC suspects (*N* = 50000)**	**Offending prevalence**	**Recidivists (*n*)**	**Incident costs^a^**	**Lifetime costs^b^**	**Lifetime costs (with QALY)^c^**
Future contact offending	Lower bound: 2% (Seto et al., 2011)	1,000	£16,172,000	£197,535,000	£225,814,000
	Upper bound: 2.3% (Wakeling et al., 2011)	1,150	£18,597,800	£227,165,250	£259,686,100
Future internet offending	Lower bound: 3.4% (Seto et al., 2011)	1,700			
	Upper bound: 4.6% (Wakeling et al., 2011)	2,300			

a *Estimate 25% offenses at £43,214 (penetrative) and 75% at £7,158 (non-penetrative) in 2019 GBP*.

b *Estimated at £197,535 in 2019 GBP*.

c *Estimated at £225,814 in 2019 GBP*.

##### Lower Bound Estimate Based on Official Records

Given the conservative recidivism rates identified by Seto et al. (2011) it would be anticipated that 4.6% of the 50,000 suspect pool would go on to commit a new sexual offense of some kind within 1.5–6 years (*n* = 2,300). Specifically, 2% with a contact sexual offense (*n* = 1,000) and 3.4% with a further IIOC offense (*n* = 1,700). Under this model, there are potentially 1,000 future victims of contact sexual offenses. Heeks et al. (2018) annual costs can be predicted over the length of time included in follow up studies, as such, incident costs are estimated over 1.5–6 years. Combined with lifetime and lifetime (with QALY) future contact offending against 1 victim per offender could contribute a further socio-economic burden between £16 million (incident costs) and £198 million-£226 million (QALY) (incidence costs).

##### Upper Bound Estimates Based on Detection of Dual Offenders

If we assume more dual offenders amongst the suspect pool then we can apply Wakeling et al. (2011) prevalence rates. Conservative application of their consolidated prevalence rate (6.6% across all risk levels) to the national suspect pool suggests that 3,300 new offenses could occur within two years. This would be estimated to include 2,300 (4.6%) offenders committing new internet offenses and 1,150 (2.3%) offenders committing new contact offenses. Under this model, there are potentially 1,150 future victims of contact sexual offenses which with a conservative estimate of 1 victim per offender could contribute a further socio-economic burden between £18.6 million (incident costs) and £227 million–£260 million (QALY) (lifetime costs).

## Discussion

This article has presented two ways in which the socio-economic burden that is potentially contributed by child sex offenders can be estimated. The aim of this work was to demonstrate the potential scale of the problem within the UK and to provide a clear argument for evidence based policing strategies to help identify the riskiest offenders, in terms of their risk of contact offending.

In our first analysis, we consider the amount of pedophilic and hebephiliac males in the UK who are likely contact offenders. At one victim per offender, the combined socio-economic burden from males with pedophilic and hebephiliac sexual preferences could amount to somewhere in the region of £236-£597 million incident costs and £2.9-£7.3 billion lifetime costs (increased to £3.3-£8.3 billion including QALY). These figures are likely underestimates given the average number of children abused by pedophiles [estimates ranging from 1.7 for homosexual incest pedophiles to 27 for bisexual pedophiles; Abel and Harlow (2001)]. The results of our first review question suggests that targeted police action toward those offenders with pedophilic and hebephiliac themes in their online behavior is a sound investigative strategy. However, further work is needed to understand the differences between those who admit to using indecent images and those that admit to contact sexual offenses. In this review, rates of indecent image use were low (Table 2) but contact offenses were even lower. This concurs with broader meta-analysis of Seto et al. (2011) and Babchishin et al. (2014). As the work by Babchishin et al. (2014) demonstrates, IIOC only offenders have a higher rate of pedophilia than contact sexual offenders against children. Dual offenders had higher rates of pedophilia than IIOC only offenders and also pose a greater risk of contact sexual offenses. As such, investigative strategies that focus on both evidence of pedophilia, hebephilia and concomitant contact offending would appear to be the optimal approach.

In our second analysis, we examined the national pool of 50,000 IIOC offenders in the UK (CEOP, 2013) estimating that between 6,000 and 27,500 dual offenders could have already contributed to an economic burden of between £97–£445 million incident costs and £1.2–£5.4 billion lifetime costs (increased to £1.4–£6.2 billion including QALY). Given the problems in defining prevalence of past contact sexual offenses it is likely that the estimates are conservative and that there have been a larger number of victims for the highest risk cases. We know very little about how many victims are harmed by detected IIOC offenders, and virtually nothing about the victims of undetected IIOC offenders. Bourke and Hernandez (2009) found an average of 14.7 past victims for each IIOC offender entering cognitive behavior therapy in prison. Although self-report studies such as this run the risk of conflating undetected victim prevalence and have been criticized (Seto, 2013) the findings help to provide reasonable assurances that one victim per offender (for both review questions) is a conservative estimate. In terms of future contact offenses amongst the suspect pool of 50,000 IIOC offenders we estimate that there could be between 1,000 and 1,150 future contact victims. These offenses could contribute a further socio-economic burden of between £16–£18.6 million incident costs and between £198–£227 million lifetime costs (increasing to £226–£260 million including QALY). It should be stressed that these estimates are based on what would be expected based on official records. It is likely that there could be further undetected internet and contact sexual offenses, but these estimates are not available.

The results of our review have supported the argument that targeted police action toward those offenders with past sex offending histories or those who share characteristics with known dual offenders makes sound moral and fiscal sense as these offenders are likely to cause more harm to victims in real terms. There is also the potential that historical victims are identified and potentially safeguarded through targeted police action, thereby contributing further to victim safety and harm reduction goals. Whilst there is a mutual consensus that action must be taken, academics and practitioners are also beginning to appreciate that indiscriminant arrests would not be effective in reducing the scope of the IIOC problem [beyond the general deterrence purpose that they would serve, e.g., Wolak et al. (2014)]. Viewing all internet IIOC offenders as high risk for contact offending or recidivism is unworkable because the population is simply too diverse. There are also ethical implications with an indiscriminant or random arrest strategy in potentially tackling less harmful offending whilst not tackling more harmful offending. Further, if a random strategy was made public via the media and resulted in few convictions, because it did not pick out the most serious offenders or those who posed the greatest risk for recidivism, it could seriously undermine public confidence in investigative competence in this area. Our review has suggested that prioritization based on aspects of behavior that are most correlated with pedophilia, hebephilia and dual offending is an effective strategy for identifying offenders who pose the greatest risk of harm to children. Indeed, the application of Wakeling et al. (2011) figures are persuasive here. If, by nature of prioritizing dual offenders, law enforcement agencies detect more dual than internet only offenders more victims can be safeguarded. Here, even a modest estimate of 150 additional victims saves millions in socio-economic terms along with the untold toll on victims' lives.

In terms of practical implications for law enforcement, our review demonstrates the importance of evidence-led risk prioritization tools. Currently, only one such tool has been developed and validated in investigative contexts. KIRAT (Long et al., 2016) prioritizes individuals suspected of possessing, making, taking and/or distributing IIOC based on their risk of *also* committing contact offenses. KIRAT focuses on previous criminal history, access to children, current online and offline behavior, and other relevant factors, and shows a 95% accuracy rate for high-risk offenders and a 20% false positive rate for lower risk offenders. Prior to KIRAT, the relative significance of risk variables were unknown – and over 150 variables identified by the research team that many officers might think discriminate between contact and IIOC offenders have now been discarded. It is the national standard in the United Kingdom, having been legally mandated for use in all 43 UK police forces by the National Police Chiefs Council. KIRAT has many benefits for police force; it is simple to use, does not require clinical training, it is objective and immune to offenders' forensic awareness since it is based largely on criminal history rather than indecent image preference or search terms. Further work is needed to develop KIRAT and to help embed and sustain use within police forces internationally. A further promising tool, CPORT has been developed by Michael Seto and colleagues (Seto and Eke, 2015; Eke et al., 2019) however this recidivism tool would benefit from further validation in investigative contexts. At present, its approach is predominantly risk assessment (who will go on to reoffend) rather than risk prioritization (who is likely to have already committed contact offenses). Our review also demonstrates the importance of technology driven strategies that prioritize offenders evidencing pedophilia, hebephilia along with concomitant contact offending. Whilst academics are working with police forces to identify suitable tools further academic research is needed to understand the links between online behavior and risk of contact offending. Existing research demonstrates language has limited potential to differentiate different types of offenders (e.g., Broome et al., 2018) and the evidence base around indecent image content and collections has proven inconclusive (e.g., Long et al., 2016).

In addition to practical implications, this work contributes to the Evidence Based Policing literature on cost effectiveness. Economic Evaluation is one of the five key aspects of policing intervention evaluation [as outlined in the College of Policing's EMMIE model; Thornton et al. (2019)] and academic-practitioner partnerships are working in multi-disciplinary settings to explore a range of ways by which the cost effectiveness of police action can be assessed [Crime Harm Index, Sherman et al. (2016); QALY, Heaton and Tong (2016)]. Our paper is the first of its kind to provide an economic framework that could be applied to establish the cost effectiveness of a range of evidence-led strategies to tackle online child sexual abuse. As such, we anticipate that this paper will be of interest to a range of academics and practitioners across multiple disciplines.

There are a number of limitations with our approach in the current study. First, we rely on single studies as the basis of estimates rather than meta-analyses. However, there are limited systematic reviews and meta-analyses available. Some of the available evidence is based on small, non-representative, convenience samples, age restricted samples, broad range of measures and definitions. We have attempted to identify those studies that have large population based national samples of community males from a variety of ages and which utilize some discriminate operationalization of sexual interest. These could be improved upon and we recognize that our choices may impact the validity of our findings. Specifically, Bartova et al. (2020) ask whether respondents “have such a preference” for intimate contact with pubescent or pre-pubescent children. This does not help to establish how exclusive that preference was. Bartova et al. (2020) approach falls foul of the types of criticisms levied at surveys by Seto (2013). Dombert et al. (2016) methodology takes us closer to understanding exclusive and non-exclusive pedophilic types but they did not include hebephilic sexual interests and so were not utilized in this study. A second limitation is the application of pedophilic individuals' contact offending prevalence to males with hebephilia. Further population-based studies aligning more closely to DSM-V criteria (including contact behavior and mirrored content for hebephilia) would be clearly beneficial here. One final limitation is the application of pedophilia and hebephilia prevalence to the male population aged 16–17 years of age. The studies cited in Table 2 had surveyed males over 18 years of age. We made the decision to estimate national prevalence from 16 years as males of this age can be diagnosed with pedophilia and pedophilic disorder. And yet, we do not know the extent to which prevalence and rates of contact offending apply to this younger group. Set against these limitations, the rates of pedophilia and hebephilia reported here are within the range of those reported by other researchers (e.g., Seto, 2013) and so we are confident that we have not radically overestimated prevalence of pedophilia and hebephilia. Further work is needed on contact offending prevalence and, in the absence of UK studies, it would be helpful to establish the external validity of international studies.

There are a number of limitations with our cost estimates. First, our estimates assume that victims disclose their abuse and so use the services making up the bulk of tangible costs. However, as few as 14% of sexual violence victims report offenses to the police (Daly and Boughours, 2010). Further work is needed to understand economic costs attributable to non-reported offenses. Certainly, the innovative approaches being developed to capture intangible costs would be insightful here, as these costs would apply whether a victim disclosed or not. Second, Heeks et al. (2018) do not specifically identify costs of sexual violence with child victims. As we have noted above, we anticipate this has led to cost underestimation but further work examining child related costs would be clearly beneficial. Third, we assume an equivalence between prevalence based annual costs and cost per incident. This is not inconsistent with the economics literature but we should be categorically clear that our incident estimates do not purport to be annual estimates. What we take from this prevalence based approach is the cost that could be anticipated from each sexual offense and apply this as a cost per incident, irrespective of time frame. Fourth, the evidence underlying assumptions in Letourneau et al. (2018) is essentially US centric. Further and more detailed work would be needed to interrogate the equivalent literature base in the UK, drawing upon databases routinely used within Health Economics as well as Psychology. Fifth, whilst Letourneau et al. (2018) approach is particularly in-depth some measures are under developed. For example, educational impacts are measured using the typical costs of special education and this does not do justice to the profound educational impacts following sexual violence (e.g., Bolger, 2016). Further, Letourneau et al. (2018) estimates are based on victimization at age 11 years. This is a good starting point given the age preferences of males with pedophilia and hebephilia but further work is needed to explore variation in victim costs as a function of victim age. Finally, we apply costs to each estimated past or future contact offense and this is despite there being a wide range of contact offenses amongst males with pedophilia, hebephilia and dual offenders. Cohen and Galynker (2002), for example, found that pedophilic individuals are 2.5 times more likely to engage in physical contact with a child than voyeuristic or exhibitionistic activities. However, in a review article, Hall and Hall (2009) report that pedophilic individuals typically engage in fondling and genital manipulation more than intercourse. Exceptions to this include cases of incest, hebephilia and when children are physically coerced (Murray, 2000; Cohen and Galynker, 2002). We have tried to address this, to some extent, in the application of Heeks et al. (2018) by estimating only 25% of cases involve penetrative acts of rape. However, we acknowledge further scrutiny would be beneficial here.

There are a number of ways in which the present study could be developed. Further work exploring victimization would be beneficial. For example, Abel et al. (1987) found that pedophilic individuals with male victims had a higher number of victims. A more realistic assessment of victim numbers would drastically alter the estimates provided here. Further, conspicuously absent from this review is the population of contact sexual offenders who are neither exclusively pedophilic nor hebephilic and yet pose a considerable risk of harm to children. In Seto (2009) study only 50% of child sexual abuse offenders expressed a sexual preference for children. This population contributes significant socio-economic burden and yet it was not deemed feasible to gain prevalence and rates of contact offending from the population-based approach taken in the present study. Investigating authorities are also beginning to appreciate the considerable impact of “high harm” offenders; those online offenders who have little intention of meeting children in real life but coerce children to engage in sexual acts through blackmail or coercion. Evidence is beginning to emerge on the impacts of online child sexual abuse and how it can engender levels of sexual harm experienced by online victims comparable to that experienced by offline victims of child sexual offenses (Hanson, 2017; Hamilton-Giachritsis et al., 2017; Jonsson et al., 2019). Any measure of national socio-economic burden needs to take account of this emerging group. Further development is also needed in establishing the vicarious costs associated with being a victim of an IIOC offense. In this study, we estimate that there could be between 1,700 and 2,300 future IIOC offenses but it is not possible, as yet, to attach a meaningful cost to these offenses in terms of victim harms. Evidence is beginning to emerge that highlights the additional impact that IIOC distribution has on its victims. Many victims report that additional distress is caused by knowing that images of their abuse are circulated and viewed for sexual purposes (von Weiler et al., 2010) leading some authors to conclude that this constitutes revictimization in real terms as victims experience exacerbated PTSD symptoms including flashbacks and panic attacks. The extent to which IIOC internet offenders are made culpable for prolonged victim distress, by downloading and sharing IIOC, is being hotly debated in the US Supreme Court. Certainly, we might expect in time that the human cost of being a victim during the commission of a sexual offense for IIOC purposes is more realistically calculated. With further work, such as that started by Saied-Tessier (2014) and through restitution awards we might gain further clarity about how to calculate victim costs for IIOC offenses.

There is a more general point to be made here about wider community costs. Researchers and legal academics argue that, in very simple terms, social harms arise from the sexual objectification of children for adults' sexual pleasure. Leary (2007) argues that the vicarious harm emerging from IIOC images manifests in many ways; offenders may use images for sexual gratification, to groom children to be sexually molested, to support the idea that adult-child sexually abusive relationships are acceptable, to decrease the inhibitions of potential victims, to demonstrate to victims how to sexually please a sexual offender, to control victims, to barter/exchange on the internet, and for profit (pg. 13). In short, IIOC images create additional harm and costs to children not in IIOC images and to society in general. Whilst it is difficult to attach a socio-economic metric to this it does point toward the vicarious harms that the proliferation of IIOC online may potentially cause, further justifying evidence based and targeted policing strategies in this area.

Whilst the cost estimates we present here are alarming our intention is not to provoke moral panic, particularly in the discussion around persons with pedophilia and hebephilia. Our cost estimates are based on the assumption that the overwhelming majority of individuals with these sexual preferences (96.2–98.5%) do not contact offend. Rather, our intention is to outline the very real risk that police offices and other public sector agencies work with every day, and which impact on the safety and safeguarding of children. As such, a key issue, in our view, are evidence-based methods to assist in the triaging of this otherwise overwhelming task. The estimates that we provide should be seen as a “starting point” of a much wider discussion about the benefits of police action in this area and how these benefits may be measured. We argue that evidence-led decision criteria can start to facilitate investigative goals by helping to prioritize high risk offenders and in doing so, contribute most successfully to harm reduction. It is hoped that by outlining potential impact in socio-economic terms that funders and policy makers would continue to recognize the fiscal and moral value of funding the collaboration between academics and police forces as they attempt to make the most efficient use of limited public sector resources.

With more detailed and focused cost estimates (as outlined above) we can start to produce a sketch of how the economic and social burden of targeted and random approaches can be calculated. This could take the form of quasi experiments where police forces who take targeted approaches Vis “business as usual” or random approaches can be compared. Alternatively the implementation of targeted approaches can be staggered so as to provide a baseline of “business as usual” or random approaches for police forces in the period before targeted approaches are rolled out (Bandyopadhyay, 2017). Sufficient time will be needed to establish legal outcomes, that is, whether targeted approaches did, (a) identify individuals with more historical contact offenses, and related to this then (b) safeguard more historical victims and lower potential rates of victimization and offending, and potentially (c) contribute to higher rates of conviction with (d) longer sentences (as per crime harm index calculations). It may be the case that targeted approaches help lower offending rates because of deterrence and improved apprehension may deter more would be offenders with consequent benefits for potential victims. It is necessary to establish how much of the outcomes can be attributed to targeted approaches as concurrent interventions as well as socio-economic and technological change within the same time frame can all impact the outcomes we are trying to measure. The cost of incorrect identification of offenders would also need be taken into account.

It is clear, given the exponential rise of IIOC online, that action must be taken now to offset the potential long term problems we might witness by failing to address the problem before it gets worse. For example, the proliferation of IIOC on the internet could stretch police forces beyond their investigative capabilities and could lead to desensitization to IIOC images. The widespread availability of IIOC could promote trivialization of content and thereby encourage further offending (e.g., Quayle, 2008). A further concern is that online pedophilic individuals will continue to gain social cohesion, positive reinforcement and validation for their identities whilst offending online (Brennan and Hammond, 2011). Finally, children may be able to access IIOC more easily (e.g., Koontz, 2004; Dombroski et al., 2007) which raises concerns for indirect victimization or desensitization to IIOC related themes.

Our aim has been to estimate the potential scale of the problem in the UK to help understand the need for continued targeted action. As our analysis stands we suspect that we may have underestimated rather than overestimated the scale of the problem. Whilst we acknowledge limitations with the assumptions underlying our estimates these figures are a starting point for future discussion. We encourage feedback from others about how we might develop our calculations.

## Data Availability Statement

The original contributions presented in the study are included in the article/supplementary material, further inquiries can be directed to the corresponding author/s.

## Author Contributions

SG undertook literature searches and reviews, she conducted the economic analysis and prepared the paper for publication. LA identified the need for the research and worked with policing colleagues to help establish the review questions and contributed to various drafts of the paper. Both authors contributed to the article and approved the submitted version.

## Conflict of Interest

The authors declare that the research was conducted in the absence of any commercial or financial relationships that could be construed as a potential conflict of interest.

## Footnotes

^1^Available online at: https://www.ons.gov.uk/peoplepopulationandcommunity/populationandmigration/populationestimates (accessed September 1, 2020).
